# Parastomal Hernia Prevention With Mesh in the Context of Laparoscopic Approach: An Opinion Based on Current Literature

**DOI:** 10.3389/fsurg.2018.00019

**Published:** 2018-03-06

**Authors:** Manuel López-Cano, José Antonio Pereira Rodriguez

**Affiliations:** ^1^Department of General Surgery, Abdominal Wall Surgery Unit and General and Digestive Surgery Research Group, Institut de Reserca Vall d’Hebron (VHIR), Hospital Universitari Vall d’Hebron, Universitat Autònoma de Barcelona, Barcelona, Spain; ^2^Servei de Cirurgia General, Hospital del Mar, Parc de Salut Mar, Departament de Ciències Experimentals i de la Salut, Universitat Pompeu Fabra, Barcelona, Spain

**Keywords:** laparoscopy, parastomal, hernia, prevention, mesh

## Introduction

Since the 1990s, laparoscopic surgery (LS) has been a true revolution in the field of surgery. LS decreases the lesions inherent in surgical access modalities, reducing operative morbidity and reaching similar or even better results than those related to open surgery (OS) (1). Nowadays, surgical specialties related to stoma formation (i.e., general surgery and urology) routinely incorporate LS as a surgical approach modality. However, despite this minimal access approach, the incidence of a parastomal hernia (PH) remains high and can vary depending on the type of stoma. In this way, the frequency of PH associated with an end-colostomy ranges from 4 to 93%. This disparity can be explained by the diagnostic method used. Thus, when the diagnosis is clinical, the values range from 4 to 48% (2), and when it is radiological, from 78 to 93% (3, 4). PH figures associated with an end-ileostomy between 2 and 28% have been described (5) and when associated with an ileal conduit diversion can be up to 29% (6). The lack of a uniform PH definition (clinical or radiological) may explain the difficulty of quantifying the exact incidence of this pathology. Independently of the surgical approach (open or laparoscopy) PH repair is notoriously difficult (7) and surgical research has started to focus more and more on the prevention of PH formation by using mesh at the time of stoma construction in the context of both open and laparoscopic approaches (8).

Interestingly, in parallel with LS and in response to limitations in the understanding and use of published evidence, evidence-based medicine (EBM) began as a movement in the early 1990s and integrates clinical judgment, recommendations from the best available evidence and the patient's values (9). Knowledge of the “best available evidence” necessarily requires an understanding of study design hierarchy. The reason for which studies are placed into a hierarchy is that those at the top are considered the “best evidence”, which allows the establishment of a recommendation for practice (10). In general, there are different systems to rate the quality of evidence (high-quality evidence rated as “1” or “high” and low-quality evidence rated as “4 or 5” or “low”) (11). It is not the aim of this document to provide an analysis of the systems that can be used to place a study into a hierarchy and, depending on the system, to place the study at a different “level”. Available literature on PH prevention with mesh in the context of laparoscopic approach can be broadly categorized as those studies of an observational nature (“low quality of evidence”) and those studies that have a randomized experimental design or are meta-analyses of randomized controlled trials (“high quality of evidence”). The aim of this document is to review the studies present in MEDLINE (Pubmed) related to PH prevention with mesh in the context of laparoscopic approach (main procedure and/or mesh placement) and by stoma type (i.e., end-colostomy, end-ileostomy, and ileal conduit diversion) from the “lowest” to “highest” quality. (Search terms- “ostomy”, “end-colostomy”, “end-ileostomy”, “ileal conduit diversion”, “laparoscopy”, “prophylaxis”, “prevention”, “surgical mesh”, “prosthesis”, “implant”, “parastomal hernia”). Table 1 presents a summary of the included studies.

**Table 1:** Summary of studies on PH prevention with mesh in the context of laparoscopic approach.

**Author, Year**	**Study type**	**Surg type**	**Stoma type**	**N**	**N Lap**	**N Open**	**Mesh position**	**Mesh type**	**F-U (Mean)**	**Mesh compli**	**PH WithMesh**	**PHWithout Mesh**	**Conclusion**
**Observational studies**													
Berger, 2008 (12)	Observ	Elect	End colos	25	6	19	IPOM (Keyhole)	PreformedPVDF-DynameshIPST^®^	11 months	No	0/25	–	Prophylactic mesh is safe and effective in the short run
Janson, 2010 (13)	Observ	Elect	End colos	25	25	–	Retrom (keyhole)	large-pore, low-weightUltrapro^®^	19 months	3	3/25	–	Easy-safe procedure associated with a low rate of PH
Martínek, 2012 (14)	Observ	Elect	End colos	4	4	–	IPOM (Keyhole)	PreformedPVDF-Dynamesh IPST^®^	6 months	No	0/4	–	safe and effective procedure with a potential to reduce the risk of PH
Hauters, 2012 (15)	Observ	Elect	End colos	20	17	3	IPOM (Sugerbaker)	Preformed Largepore polyesterMesh.Parietex^®^	24 months	1	1/20	–	IPOM reinforcement in selected patients is a very promising procedure
Williams, 2015 (16)	Pilot Case-Control	Elect	End-colo and end-ileo	22	4	18	SMART technique(keyhole)	CollagenPermacol^®^	21 months	No	4/22	–	clinically safe but randomised controlled trials are required to determine its efficacy in reducing PH
Valdés-Hernández, 2105 (17)	Observ	Elect	End colos	45	6	39	Retrom (keyhole)	Polypropylene low weight	22 months	No	3/45	–	prophylactic polypropylene mesh is safe and feasible
Köhler, 2016 (18)	Observ	Elect	End colos and end ileos	80	57	23	IPOM (Keyhole)	PreformedPVDF-DynameshIPST^®^	21 months	No	3/80	–	Prophylactic mesh is safe and effective
Hauters, 2016 (19)	Observ	Elect	End colos	29	24	5	IPOM (Sugerbaker)	Preformed Largepore polyesterMesh.Parietex^®^	48 months	No	2/29	–	Prophylactic mesh according modifiedSugarbaker is an effective technique
**Randomized controlled trials**													
López-Cano, 2012 (4)	RCT	Elect	End colos	34	34	–	IPOM (Keyhole)	Composite meshProceed^®^	12 months	No	1/18	3/16	prophylactic mesh by a purely laparoscopic approach reduced the incidence of PH
Fleshman, 2014 (20)	RCT	Elect	End colos and end ileos	113	38	75	Retrom (keyhole)	CollagenStrattice^®^	24 months	3	5/55	7/58	Prosthetic mesh was safe, but it did not significantly reduce the incidence of PH
Vierimaa, 2015 (21)	RCT	Elect	End colos	66	66	–	IPOM (Keyhole)	Flat meshDynaMesh-IPOM^®^	12 months	–	12/32	17/32	mesh does not reduce the radiologically PH.Mesh reduce clinically PH
Jánó, 2016 (22)	RCT	Elect	End colos	84	22	62	3D impl.Around stoma(Keyhole)	Preformed polyp. Mesh Surgimesh Parastomal^®^	19.2 months	No	3/38	18/46	Ongoing trial/interim analysis
López-Cano 2016 (23)	RCT	Elect	End colos	52	52	–	IPOM (Sugerbaker)	Composite meshPhysiomesh^®^	12 months	No	6/24	18/28	mesh by the lap approachmodified Sugarbaker technique is safe and effective in the prevention of PH

## End-Colostomy

### Observational Studies

Observational studies (ObS) are clinical research designs whose goal is the observation and description of events without any intervention in their natural course. ObS represent 80% of the publications in biomedical journals, independent of the database indexing and eventual impact factor of each journal (24). Most of the studies we found on PH prevention with mesh in the context of laparoscopic approach were observational and relating to end-colostomy construction in an elective setting (12–19). Apart from the inherent methodologic limitations of ObS that generate bias (25), the studies we found comprise a limited number of patients (143 patients), and the results related to laparoscopic approach are indirect, because six of eight studies (12, 15–19) combined the open and laparoscopic approach. Regarding mesh position, the studies are heterogeneous: in three studies the mesh position is an intraperitoneal onlay (IPOM) with a gap in the middle of the mesh (Keyhole) (12, 14, 18), in two a retromuscular position with a keyhole mesh (13, 17), in two an IPOM modified Sugarbaker technique (15, 19), and in one study the authors use their own mesh position they call SMART (16). The type of mesh used in this ObS is mostly a synthetic non-absorbable mesh (12–15, 17–19) and in only one study the prosthetic material is absorbable and of biologic origin (16). Additionally, the material architecture is heterogeneous: in three studies the authors use a prefabricated square mesh device with a central funnel-shaped cannel (12, 14, 18), in two a round composite mesh with a central band protecting the bowel from erosion (15, 19), and in three a flat mesh (13, 16, 17). On the other hand, in all ObS related to an end-colostomy (12–19), the follow-up was 2 years or less, and at this point, it is necessary to remember that the time elapsed since the formation of the stoma may be an important factor in relation to the onset of a PH, since although it appears that 50% of the PH will be diagnosed in the first 2 years of follow-up, the risk may continue for at least 20 years (26). All ObS related to an end-colostomy had a positive conclusion in favor of the use of a prophylactic mesh in terms of safety and efficacy.

### Randomized Controlled Trials (RCTs)

Probably, well-conducted RCTs are the best type of study for determining whether there is a causal relationship between intervention and effect (27). Although RCTs are the gold standard with regard to level of evidence, the extent to which their results can be extrapolated to the wider patient population (i.e., generalizability, external validity) is questionable because standardized and controlled study conditions do not adequately reflect the clinical reality. In the previous context real world evidence (i.e., registries) have been advocated as the best way to monitor the effects of a treatment or intervention long-term, as in the case of prevention with a medical device (i.e., mesh) (28). However, both types of data (i.e., registries and RCTs) should be complementary in the total product life cycle (i.e., preventive mesh) evaluation (29). To the best of our knowledge, no data derived from registries are present in the literature related to PH prevention with mesh in the context of laparoscopic approach. We found five RCTs regarding PH prevention with mesh in the context of laparoscopic approach (4, 20–23). Similarly to the aforementioned ObS, the RCTs found comprise a limited number of patients (212 patients) and only three studies exclusively analyze patients operated on by laparoscopic approach (4, 21, 23). Regarding mesh position, no uniformity was followed because two used an IPOM keyhole position (4, 21), one a retromuscular keyhole (20), one a 3D implant around stoma (22), and one an IPOM modified Sugarbaker technique (23). Also, in all RCTs (4, 20–23) the follow-up was 2 years or less, and the type of mesh was heterogeneous, including biological (20) and synthetic non-absorbable meshes (4, 21–23). Furthermore, the mesh used in one RCT (23) has been withdrawn from the market (30). Regarding conclusions, two studies state that PH prevention using a laparoscopic approach is safe and effective (4, 23), in one study the mesh was clinically effective but radiologically ineffective (21), in one the conclusion was that the mesh did not significantly reduce the incidence of PH (20), and finally, one of the RCTs was an interim analysis, and no statistical analysis was performed (22).

### Meta-Analysis

Alongside high-quality RCTs with a low risk of systematic error (bias), meta-analyses of these provide the highest level of evidence (9). Different meta-analyses have been published in connection with PH prevention with mesh. However, it is not our intention to analyze all of them. We selected only one (8) because, to the best of our knowledge, this meta-analysis is the only one that includes a trial sequential analysis (TSA), and TSA is a statistical tool recommended for inclusion in a meta-analysis (31). TSA is a methodology that combines an optimal information size (OIS) calculation for a meta-analysis with the threshold of statistical significance (statistical reliability of data), controls the risk of type I errors (false-positive results), and helps to clarify whether additional trials are needed in the topic under study. The conclusions of this meta-analysis (8) were that PH prevention with a permanent synthetic mesh, in a retromuscular position, when creating an end-colostomy by an open approach significantly reduces the incidence of PH and the risk for subsequent PH repair and does not increase surgical site infections. The reduction in PH incidence is more pronounced when only clinical follow-up is done compared with systematic CT scan follow-up. TSA shows that the OIS is reached for the primary outcome (PH prevention), and additional RCTs in the previous context are not needed. More data are needed to increase precision and obtain firm evidence regarding PH repair reduction, the low rate of surgical site infections, and the effectiveness of laparoscopic approach.

## End-Ileostomy and Ileal Conduit Diversion

Regarding end-ileostomy, only two observational studies (16, 18) and one RCT (20) included patients with this type of ostomy in the context of laparoscopic approach. The number of patients was very limited, and no comments can be made in relation to this type of ostomy. No studies were found about PH prevention with mesh in the context of laparoscopic approach in connection with an ileal conduit diversion.

## Summary

Based on the current data, PH prevention with mesh in the context of laparoscopic approach is an unresolved issue. Most research is observational with positive findings and conclusions in favor of the use of a prophylactic mesh in terms of safety and efficacy (12–19). However, the inability to attribute causation (a fundamental limitation of observational research) was rarely mentioned in different papers. A possible consequence of inadequate reporting of limitations of observational studies is that readers consider the reported associations to be causal, promoting preventive practices based on evidence of modest quality. Few research studies on PH prevention with mesh in the context of laparoscopic approach have been based on RCTs (4, 20–23), and this research is heterogeneous with no uniform conclusions and unable to give a general recommendation. Furthermore, to the best of our knowledge, no data derived from registries are present in the literature. Data derived from meta-analyses (8) reveal with firm evidence that PH prevention with a permanent synthetic mesh, in a retromuscular position, when creating an end-colostomy by an open approach significantly reduces the incidence of PH. However, more data are needed to increase precision and obtain firm evidence regarding laparoscopic approach. In conclusion, more data are needed in the form of well-designed observational studies, RCTs, and registries on PH prevention with mesh in the context of laparoscopic approach (main procedure and/or mesh placement).

## Author Contributions

MLC designed the study, reviewed the literature, wrote the paper, and prepared the final draft. JAP reviewed the manuscript and approved the final draft.

## Conflict of Interest Statement

The authors declare that the research was conducted in the absence of any commercial or financial relationships that could be construed as a potential conflict of interest.
